# Binning long reads in metagenomics datasets using composition and coverage information

**DOI:** 10.1186/s13015-022-00221-z

**Published:** 2022-07-11

**Authors:** Anuradha Wickramarachchi, Yu Lin

**Affiliations:** School of Computing, Australian National University, Canberra, Australia

**Keywords:** Metagenomics binning, Long reads, Machine learning, Clustering

## Abstract

**Background:**

Advancements in metagenomics sequencing allow the study of microbial communities directly from their environments. Metagenomics binning is a key step in the species characterisation of microbial communities. Next-generation sequencing reads are usually assembled into contigs for metagenomics binning mainly due to the limited information within short reads. Third-generation sequencing provides much longer reads that have lengths similar to the contigs assembled from short reads. However, existing contig-binning tools cannot be directly applied on long reads due to the absence of coverage information and the presence of high error rates. The few existing long-read binning tools either use only composition or use composition and coverage information separately. This may ignore bins that correspond to low-abundance species or erroneously split bins that correspond to species with non-uniform coverages. Here we present a reference-free binning approach, LRBinner, that combines composition and coverage information of complete long-read datasets. LRBinner also uses a distance-histogram-based clustering algorithm to extract clusters with varying sizes.

**Results:**

The experimental results on both simulated and real datasets show that LRBinner achieves the best binning accuracy in most cases while handling the complete datasets without any sampling. Moreover, we show that binning reads using LRBinner prior to assembly reduces computational resources required for assembly while attaining satisfactory assembly qualities.

**Conclusion:**

LRBinner shows that deep-learning techniques can be used for effective feature aggregation to support the metagenomics binning of long reads. Furthermore, accurate binning of long reads supports improvements in metagenomics assembly, especially in complex datasets. Binning also helps to reduce the resources required for assembly. Source code for LRBinner is freely available at https://github.com/anuradhawick/LRBinner.

**Supplementary Information:**

The online version contains supplementary material available at 10.1186/s13015-022-00221-z.

## Introduction

Metagenomics binning is an important area of study in metagenomics analysis. Broadly, metagenomics enables the study of microbial genetic material directly from the source environment [1]. This eliminates the necessity of lab culturing thus revealing the microbial content of an environment as it is without culturing biases. *Metagenomics binning* is one key problem in metagenomics studies that facilitates the clustering of sequences into different taxonomic groups. Mainly there are two approaches to address this problem; (1) reference-based binning and (2) reference-free binning. Reference-based binning tools (e.g., Kraken [2], Centrifuge [3] and Kaiju [4]) bin sequences based on similarity by comparing with a database of known reference genomes and thus face challenges when the reference database is unavailable or incomplete. At present, reference-free binning tools have been gaining popularity over reference-based binning tools, especially in discovering novel or rare species in complex metagenomics datasets. While Next-Generation Sequencing (NGS) technologies produce short reads, existing reference-free binning tools typically rely on longer *contigs* that are assembled from short reads and contain richer information for binning. Reference-free binning tools (e.g., MetaBAT [5, 6], MaxBin [7, 8], BMC3C [9], BusyBeeWeb [10, 11], SolidBin [12], MetaProb2 [13] and VAMB [14], etc.) bin contigs based on their composition and coverage information, without using any reference database. For example, a recent work VAMB [14] introduced the use of deep variational auto-encoders to perform reference-free unsupervised binning of contigs incorporating both the composition and coverage information. VAMB then uses an iterative medoid clustering algorithm which extracts clusters (bins) in a local search fashion. Thanks to the accurate composition and coverage information of contigs, reference-free approaches show promising results in binning contigs from metagenomics assemblies.

With the advent of Third Generation Sequencing (TGS) technologies such as Pacific Biosciences (PacBio) and Oxford Nanopore Technologies (ONT), reads obtained are much longer than NGS reads (>10kbp). Therefore, more information becomes available in the reads themselves to support direct reads binning. However, contig-binning tools cannot be directly applied to bin long reads (by treating them as contigs) because there is no coverage information available for each long read. Moreover, while certain contig-binning tools make use of single-copy marker genes to estimate the number of bins in the sample, the high error rates of long reads and the varying coverages of different species make it infeasible to derive accurate estimations. Furthermore, datasets containing raw long reads are much larger in size compared to typical datasets containing contigs, and hence, demand more scalable reference-free binning tools. Recently, a long-read binning tool named, MetaBCC-LR [15] was introduced to bin error-prone long reads. While MetaBCC-LR shows very promising results in binning long reads, it still suffers from accuracy and scalability issues, especially in complex metagenomics datasets. Firstly, MetaBCC-LR uses the composition and coverage information of long reads in a separate manner (*i.e.*, in two different stages). This can result in the ignorance of bins for species with low abundance and incorrect bin split for species with non-uniform composition or coverage. Secondly, due to its scalability issue, MetaBCC-LR has to employ a sampling strategy to work on a subset of reads for large datasets, which affects its overall binning accuracy. MetaBCC-LR [15] showed that concatenation of coverage and composition features causes inaccuracies in clustering as the TSNE dimension reduction considers the input to be a single vector. More recently, VAMB [14] showed that for the contig coverage (computed from read alignments) can be combined with its composition successfully using deep learning followed by iterative medoid clustering. However, neither read alignments nor iterative medoid clustering are suitable to handle long-read datasets because long reads are typically orders of magnitude more than contigs. In addition, binning of long read datasets requires novel algorithms to detect clusters of vastly varying sizes (hundreds to millions of reads per species), which is different from the contig-binning scenarios (few hundreds of contigs per species [16]). Therefore, it is persistently demanding better approaches to bin massive long-read datasets accurately and efficiently. The requirement is further supported by the advent of PacBio HiFi technology [17] which produces accurate and massive long-read datasets in metagenomics studies.

In this paper, we present LRBinner to bin TGS long reads without using any reference databases. LRBinner combines the composition and coverage features and eliminates the need to sub-sample large datasets. More specifically, LRBinner uses a variational auto-encoder to obtain lower dimensional representations by simultaneously incorporating both composition and coverage information of the complete dataset, which was initially presented in contigs binning tool, VAMB [14]. LRBinner further uses a distance-histogram-based clustering algorithm that can capture confident clusters of varying sizes. LRBinner finally assigns unclustered reads to obtained clusters using their statistical profiles. The experimental results of LRBinner compared against other baselines show that LRBinner achieves better binning results on both simulated and real datasets. Moreover, we show that binning long reads by LRBinner prior to assembly helps to improve genome fraction of assemblies while reducing the memory consumption for metagenomics assembly.

## Methods

**Fig. 1:**
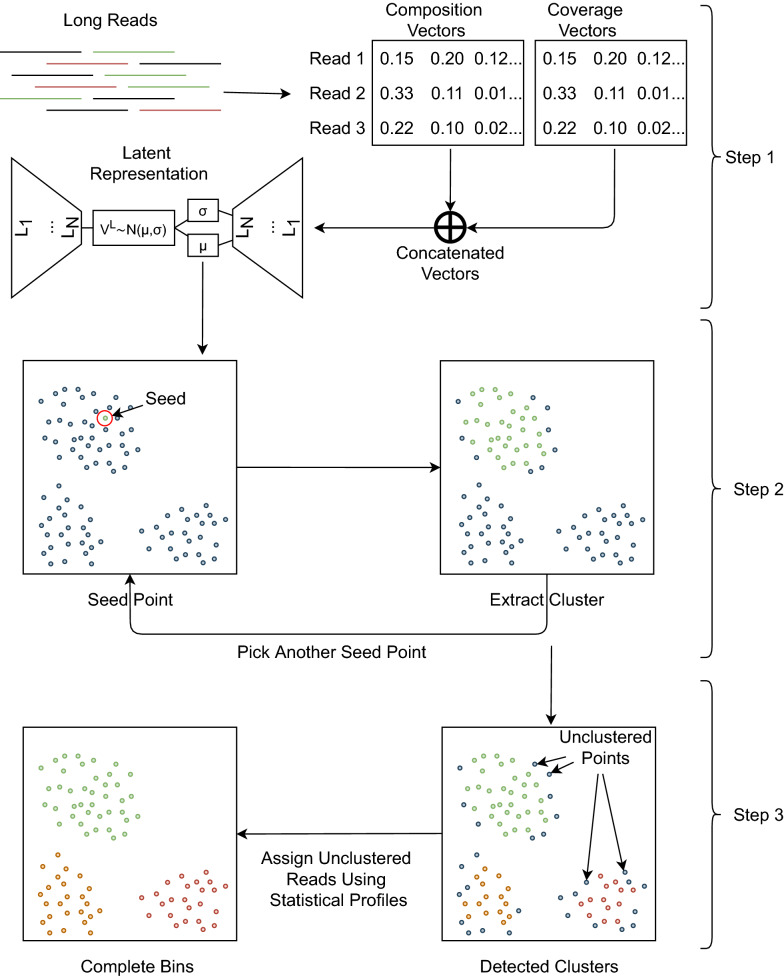
Overall workflow of LRBinner. (Step 1) The feature vectors of composition and coverage information are computed from long reads. Composition vectors are the normalized *k*-mer counts where as the coverage vectors are the normalized *k*-mer counts histograms for each read. The feature vectors are input into a variational auto-encoder to obtain low-dimensional latent representations. Note that variational auto-encoders learn a lower dimensional representation while learning to reconstruct the original. (Step 2) Sample a seed point (read) in the latent space. Use this seed to estimate a confident cluster (bin) that contains this seed point. Step 2 is iterated until there are no seed points. (Step 3) The unclustered points are assigned to the clusters using a statistical model. Note that the 2-dimensional representation of points is only for the illustration purpose

LRBinner consists of three main steps; (1) learning lower dimensional latent representations of composition and coverage, (2) clustering the latent representations and (3) obtaining complete clusters. The complete workflow for LRBinner is demonstrated in Fig. 1. In the following sections, we will explain these three steps in details.

### Step 1

LRBinner uses two typical binning features of metagenomic sequences, composition and coverage. The composition and coverage of each long read is represented as trimer frequency vectors and *k*-mer coverage histograms [15], respectively.

#### Computing composition vectors

Previous studies show that different species demonstrate unique genomic patterns [18, 19] and thus can be used in composition-based metagenomics binning. Oligonucleotide frequency vectors are one such genomic representation that can be used in metagenomics binning. Small *k*-mer sizes (*k* varying from 3-7) have been used in the past to discriminate assembled contigs of different origins [6, 8, 10, 20, 21] and 3-mers have been used in metagenomics binning of error-prone long reads [15] which shows that trinucleotide frequency vectors provide stable binning despite the noise level exist in TGS reads. Therefore in LRBinner, we utilise *k*=3 by default which results in trinucleotide composition vectors. For each long read, we count the frequencies of all 64 3-mers in this read and merge the reverse complements to form a vector of 32 dimensions. The resulting vector is then normalised by the total number of 3-mers observed in the read. We refer to this trimer frequency vector as $V^T$.

#### Computing coverage vectors

While an all-vs-all alignment of long reads may provide coverage information for each long read, it is usually too time-consuming to perform the quadratic number of pairwise alignments on large scale long-read datasets. Given a sufficiently large *k*, the frequency of a *k*-mer is defined as the number of occurrences of this *k*-mer in the entire dataset. Long reads from high-abundance species tend to contain *k*-mers with higher frequencies compared to long reads from low-abundance species. Hence, a *k*-mer frequency vector can be computed for each long read to represent coverage information without performing alignments [15] to represent read coverage. In order to obtain such coverage histograms, we first compute the *k*-mer counts of all long reads in the entire dataset by DSK [22] (the default value of *k*=15). The counts are then indexed in memory by encoding each nucleotide in 2 bits as per the encoding (*i.e.*, A=00, C=01, T=10 and G=11) [22]. The resulting index is in the form $(k_i, coverage(k_i))$ (as *key*, *value* pairs), where $coverage(k_i)$ is the number of occurrences of the *k*-mer $k_i$ in the entire dataset. Now for each *k*-mer $k_i$ of a read, we obtain the frequency from the index. These frequencies are then used to build a normalised histogram, $V^C$. We chose a preset bin width ($bin\_width$) for the histogram and obtain a vector of *bins* dimensions. By default we set $bin\_width$ $=$ $10$ and $bins$ $=$ $32$. All the *k*-mers with counts exceeding the histogram limits are added into the last index of the histogram. We also normalise the histogram by the total number of *k*-mers observed in the read.

#### Obtaining latent representations

For each long read, its coverage ($V^C$) and composition ($V^T$) vectors are concatenated to form a single vector *V* of 64 dimensions. We use a variational auto-encoder to obtain lower dimensional latent representations. The key motivation for using a variational auto-encoder is to combine coverage and composition features in an effective way. Previous work shows that a simple concatenation of coverage and composition vectors made TSNE less effective [15]. This is mainly because TSNE does not attempt to learn how to effectively combine composition and coverage features, but rather sticks with the spatial distances on concatenated features. However, the variational auto-encoder is able to learn lower dimensional representations by combining both composition and coverage features through a deep neural network.

Our implementation of the variational auto-encoder consists of two hidden-layers in the encoder and decoder. Each layer uses batch normalisation and dropout with $p$ $=$ $0.1$ during the training phase. For each input vector *V*, the auto-encoder learns a latent representation $V^{L}_{i}$, where $V^{L}_{i} \sim {\mathcal {N}}(\mu _i, \sigma _i)$. The latent representation consists of 8 dimensions. Each layer in the encoder and decoder contains 128 neurons. Similar to previous studies [14], we use *LeakyRELU* (leaky rectified linear unit function) for $\mu$ and *softplus* function for $\sigma$ layers. Note that $\mu$ and $\sigma$ represent neural network layers intended to learn the lower dimensional means and standard deviations of each read’s distribution. We use the weighted sum of reconstruction error *E* (Eq. 1, where $V_{in}$ and $V_{out}$ represents the inputs and the outputs of the auto-encoder. Note that they are the same for an auto-encoder) and Kullback-Leibler divergence [14, 23] $D_{KL}$ (Eq.2) as the loss function. $E_{cov}$ and $E_{com}$ represent reconstruction errors of coverage and composition respectively. Eq. 3 demonstrates the complete loss function used.

1 $$\begin{aligned} E= & {} \sum (V_{in} - V_{out})^2 \end{aligned}$$

2 $$\begin{aligned} D_{KL}(latent|prior)= & {} - \sum \frac{1}{2} (1+ln(\sigma )-\mu ^2-\sigma ) \end{aligned}$$

3 $$\begin{aligned} \textit{Total Loss}= & {} w_{cov}E_{cov} + w_{com}E_{com} + w_{kld}D_{KL} \end{aligned}$$

Here we set $w_{cov}$ $=$ $0.1$, $w_{com}$ $=$ $1$ and $w_{kld}$ $=$ $1/500$ as determined empirically using simulated data. The decoder output was obtained through LeakyRELU activation in order to reconstruct the scaled positive inputs. We train the auto-encoder with read batches of size 10,240 for 200 epochs. Finally, we obtain the predicted latent means of the input data from the encoder for clustering. Each point in the latent mean corresponds to the relevant read in the original input.

### Step 2

**Fig. 2:**
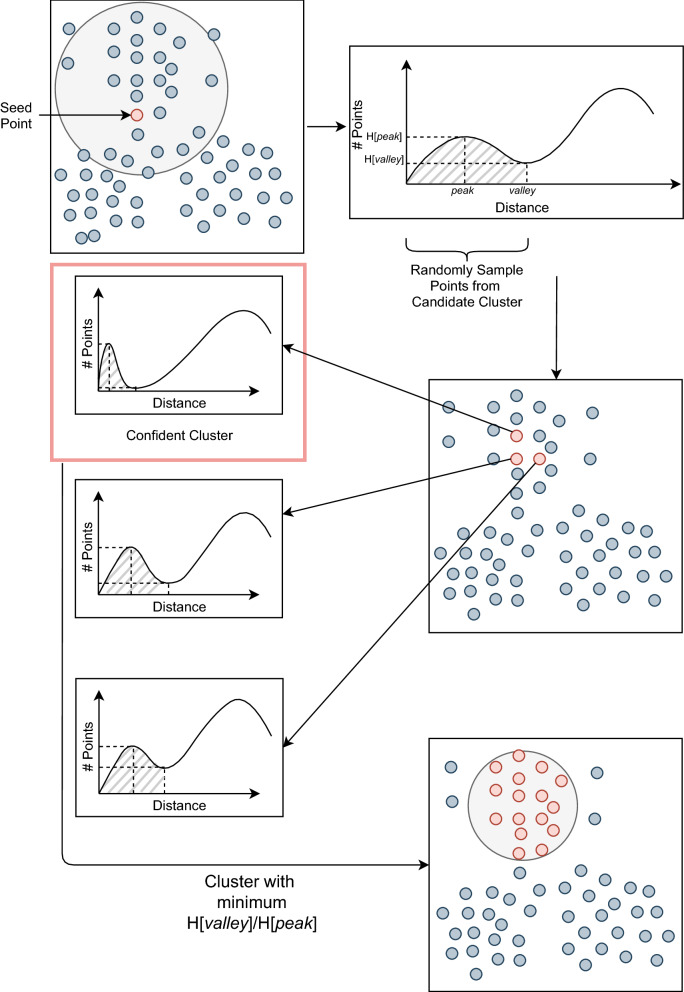
Illustration of the clustering algorithm. First, we select a seed point and compute a histogram using the distances to every other point. Then, we derive a *candidate cluster* by observing the peak of the histogram. Note that this cluster is only an estimation for the chosen point’s cluster. Sample points from the *candidate cluster* and choose a point with the minimum valley-to-peak ratio. Extract points before the *valley* to form a *confident cluster*. Valley-to-peak ratio is an indicator of density which is the highest at cluster centre. Note that the 2D representation of points is only for the illustration purposes

In this step, we perform clustering of the latent means learnt by the variational auto-encoder. The complete clustering algorithm of LRBinner is illustrated in Fig. 2. Similar to previous studies [14], we use the cosine distance as the distance measure for clustering. Note that cosine distance between point *a* and *b* in latent space $V^L$ is defined as *d*(*a*, *b*)=$\frac{V^L_a\cdot V^L_b}{||V^L_a|| ||V^L_b||}$. Given a point *a*, a distance histogram $H_a$ can be generated by computing the pairwise distances between *a* and all other points and setting the bin width as $\Delta$ ($\Delta$ $=$ $0.005$ in our experiments). We define *peak* as the index of the first maximal of the distance histogram $H_a$. Similarly, the *valley* is defined as the index of the first minimal after the *peak* in the distance histogram $H_a$. Refer to the top right figure in Fig. 2 for an example of the *peak* and *valley* in a distance histogram.

As shown in VAMB [14], a point with smaller valley-to-peak ratio *H*[*valley*]/*H*[*peak*] is more likely to be the medoid of a cluster, where *H*[*valley*] and *H*[*peak*] are the number of points corresponding to the *valley* and *peak* in the distance histogram *H*, respectively. Therefore, VAMB randomly samples points, searches within a distance of 0.05 (up to 25 neighbouring points) and moves to another point if *H*[*valley*]/*H*[*peak*] can be further reduced. This step is iterated until a local minimal point of *H*[*valley*]/*H*[*peak*] is inferred as a proper cluster medoid and then the corresponding cluster is extracted by removing points within a distance $\Delta \times valley$ of the distance histogram. However, clusters of long reads are orders of magnitude larger than clusters of contigs, thus mere local search of a cluster medoid may be inefficient. Furthermore, while most contig clusters consist of hundreds of points per species [16], the long-read clusters vary in size drastically (from hundreds of points to millions of points), which demand for a more flexible search strategy rather than sampling points within a fixed radius and up to a fixed number of neighbours. Hence, we design the following strategy to dynamically extract clusters of varying sizes. Our algorithm consists of two steps; (1) from a seed point to a candidate cluster and (2) from a candidate cluster to a confident cluster.

#### From a seed point to a candidate cluster

A point *s* is called a *seed point* if its valley-to-peak ratio $H_s[valley]/H_s[peak]<0.5$ in its distance histogram $H_s$. Initially, LRBinner randomly picks a seed point *s* from the entire dataset and obtains its distance histogram $H_s$. Note that a distance histogram demonstrates a *candidate cluster*. This *candidate cluster* consists of the points within the distance $\Delta \times valley$ in $H_s$, referred to as *candidate cluster points*. Compared to the seed point, some candidate cluster points may have lower valley-to-peak ratio that results in more confident clusters. However, the number of candidate cluster points may vary significantly depending on the size of the ground-truth clusters. In the next section, we will show how to use sampling strategies to find a confident cluster from a candidate cluster.

#### From a candidate cluster to a confident cluster

Given a *candidate cluster*, we sample 10% of candidate cluster points (up to 1,000 points) to compare their corresponding distance histograms. For each point *p* in *candidate cluster points*, we compute the valley-to-peak ratio $H_p[valley]/H_p[peak]$ in its corresponding distance histogram $H_p$. We chose a point *x* from the sample with the minimum *H*[*valley*]/*H*[*peak*] value and extract a *confident cluster* which consists of points within a distance $\Delta \times valley$ of the distance histogram $H_x$. In contrast with the iterative medoid search in VAMB [14], this approach takes advantage of the rough estimation of the *candidate cluster* from a seed point and thus allows us to dynamically and efficiently discover clusters with varying sizes. This process is iterated until no further *candidate clusters* or *seed* points are observed. Please refer to Implementation Section for detailed information. The resulting clusters are depicted as detected clusters in Fig. 1. Note that few reads still remain unclustered due to the noise present in composition and coverage vectors of error-prone long reads and we will show how to assign them to existing bins in the next section.

### Step 3

#### Obtaining final bins

Once all the clusters have been yielded, the points that are sparsely located are left aside. However, such points could have the potential to improve the downstream assembly processes. Hence, we assign such points to the detected clusters using the statistical model from MetaBCC-LR [15]. For each cluster $C_k$ the mean $\mu ^{C}_k$, $\mu ^{T}_k$ and standard deviation $\sigma ^{C}_k$, $\sigma ^{T}_k$ is computed using the coverage and composition vectors; $V^C$ and $V^T$ respectively.

4 $$\begin{aligned} PDF({\bar{v}},{\bar{\mu }},{\bar{\sigma }}) = \prod _{j}^{|{\bar{v}}|} \frac{1}{\sqrt{2\pi }\sigma _j} e^{-\frac{(x_j-\mu _j)^2}{2\sigma _j^2}} \end{aligned}$$

Finally the unclustered reads are assigned to the cluster $C_i$ using a maximum likelihood computed using Eq. 4. The assignment of reads is performed such that Eq. 5 is maximised. $V^C_l$ and $V^T_l$ are the coverage histogram and trimer frequency vectors of the unclustered read *l*.

5 $$\begin{aligned} C_i = \mathop {\mathrm{argmax}}\limits _{i} \bigg \{PDF(V^{C}_l,\mu ^{C}_i,\sigma ^{C}_i)\times PDF(V^T_l,\mu ^T_i,\sigma ^T_i)\bigg \} \end{aligned}$$

## Experimental setup

### Datasets

We evaluated LRBinner using several simulated and real datasets containing long reads. Detailed information about the datasets and constituent species are tabulated in the Additional file 1: Tables S1 and S2.

**Table 1:** Comparison of binning results of BuseBeeWeb, MetaBCC-LR and LRBinner

Dataset	Actual no. of bins	Evaluation criteria	MetaBCC-LR	LRBinner
Sim-8	8	Precision	90.78%	**99.14%**
		Recall	96.18%	**99.14%**
		F1 score	93.40 %	**99.14%**
		Bins detected	13	**8**
Sim-20	20	Precision	82.97%	**90.53%**
		Recall	81.95%	**88.23%**
		F1 score	82.46%	**89.36%**
		Bins detected	29	**18**
Sim-50	50	Precision	82.23%	**91.92%**
		Recall	70.56%	**77.03%**
		F1 score	75.95%	**83.82%**
		Bins detected	**32**	31
Sim-100	100	Precision	**90.50%**	82.60%
		Recall	84.54%	**92.78%**
		F1 score	**88.54%**	87.39%
		Bins detected	**89**	63
ZymoEVEN	10	Precision	72.41%	**91.26%**
		Recall	**92.97%**	75.36%
		F1 score	81.41%	**82.55%**
		Bins detected	**8**	17
MSA-1003	10	Precision	93.69%	**95.30%**
		Recall	95.50%	**95.99%**
		F1 score	94.59%	**95.64%**
		Bins detected	14	**10**
SRX9569057	17	Precision	**80.94**	80.47%
		Recall	85.82	**90.68%**
		F1 score	83.31	**85.27%**
		Bins detected	23	**16**
SRX9569058	17	Precision	70.18%	**73.72%**
		Recall	86.63%	**91.03%**
		F1 score	77.54%	**81.46%**
		Bins detected	37	**22**
SRX9569059	17	Precision	66.69%	**79.70%**
		Recall	73.76%	**91.25%**
		F1 score	70.05%	**85.08%**
		Bins detected	**16**	20

The best performance values and bin estimations are highlighted in bold text

**Table 2:** Comparison of assembled genome fractions, CPU time consumed for assembly and peak memory usage of assembly before and after binning the reads

Dataset	Assembly tool	Genome fraction		CPU hours		Peak memory (GB)	
		Raw	Binned	Raw	Binned	Raw	Binned
Sim-8	Wtdbg2	98.80%	**98.90%**	**0.26**	0.84	9.28	**0.96**
	MetaFlye	**99.90%**	99.85%	16.13	**11.64**	44.12	**10.65**
Sim-20	Wtdbg2	97.84%	**99.19%**	**0.16**	2.28	10.60	**0.92**
	MetaFlye	**99.80%**	99.75%	**19.44**	20.28	44.70	**11.23**
Sim-50	Wtdbg2	97.83%	**98.06%**	6.03	**5.98**	15.7	**2.68**
	MetaFlye	**99.35%**	98.43%	23.03	**20.01**	64.21	**14.58**
Sim-100	Wtdbg2	91.70%	**93.67%**	9.2	**9.1**	36.16	**10.84**
	MetaFlye	97.68%	**98.01%**	69.79	**59.89**	116.11	**27.48**
ZymoEVEN	Wtdbg2	55.17%	**58.63%**	**1.37**	1.38	11.07	**2.88**
	MetaFlye	86.51%	**86.55%**	15.17	**13.05**	31.67	**14.57**
MSA-1003$^{\dagger }$	Wtdbg2	67.45%	**82.50%**	**0.31**	1.05	23.43	**19.61**
	MetaFlye	91.40%	**91.74%**	**155.96**	158.59	62.28	**45.38**
SRX9569057	Wtdbg2	40.40%	**73.02%**	**0.26**	1.56	21.72	**3.88**
	MetaFlye	**77.73%**	73.68%	122.00	**116.20**	57.91	**26.31**
SRX9569058	Wtdbg2	37.51%	**80.65%**	**0.30**	1.98	30.79	**3.86**
	MetaFlye	79.16%	**79.63%**	**211.61**	212.58	87.62	**41.37**
SRX9569059	Wtdbg2	41.00%	**80.38%**	**0.26**	1.82	25.63	**3.80**
	MetaFlye	**79.69%**	77.46%	152.64	**129.41**	62.62	**30.56**

$\dagger$ Genome fraction computed from species with at least 0.1% abundance

The best performance values and bin estimations are highlighted in bold text

#### Simulated datasets

We simulated four datasets using SimLoRD [24] to evaluate the performance of our method. The datasets consist of 8, 20, 50 and 100 species. These datasets are named as **Sim-8**, **Sim-20**, **Sim-50** and **Sim-100** respectively. We set the average read length to be 5,000bp with default error model of SimLoRD (insertion probability=0.11, deletion probability=0.04 and substitution probability=0.01).

#### Real datasets

In order to evaluate LRBinner, we used several real datasets with known ground-truth references. To determine the origins of the reads in these datasets, the reads were mapped to the respective reference species using Minimap2 [25]. The information about the datasets are as follows.

- Reads from ZymoEVEN Mock Microbial Community with Oxford nanopore reads from NCBI Accession Number *ERR3152364* [26]. We removed shorter reads (less than 1000bp) and reads that did not align with any of the reference species in the mock community for evaluation purposes.
- Reads from ATCC MSA-1003 Mock Microbial Community with PacBio CCS reads from NCBI BioProject number *PRJNA546278* (**MSA-1003**). For the evaluation we used the top 10 species which have more than 1% abundance.
- PacBio-HiFi reads obtained from NCBI BioProject number *PRJNA680590*. There are 3 read samples (NCBI BioSample number *SAMN16885726*) and each sample consists of 21 strains for 17 species as follows;
  - **SRX9569057**: Standard input library
  - **SRX9569058**: Low input library
  - **SRX9569059**: Ultra low input library (PCR amplified sample)

### Tools for benchmarking

There is a limited number of tools that support binning of long reads. Remind that most contig-binning tools cannot be directly applied to bin long reads (even for highly accurate PacBio HiFi reads) because there is no coverage information available for each long read. Hence, in our evaluation we benchmark LRBinner against MetaBCC-LR [15] which support large error prone long-reads. Note that, BusyBeeWeb only supports up to 200MB of FASTA formatted data. Hence, we omit BusyBeeWeb from our evaluations although it can handle a smaller amount of long reads for a fair comparison.

### Evaluation criteria

In our evaluation we report precision (Eq. 6), recall (Eq. 7) and F1-score (Eq. 8) of binning. We transform the binning result to a matrix *a* of size $K\times S$, where *K* denotes the number of bins and *S* denotes the number of species. Note that $a_{ks}$ denotes the number of reads assigned to bin *k* with ground truth species *s*. In order to evaluate the quality of binning, we used AMBER [27] to obtain the completeness (defined as $\frac{\textit{true positives}_b}{\textit{true positives}_b+\textit{false negatives}_b}$ for each bin *b*) and contamination (defined as $1-\frac{\textit{true positives}_b}{\textit{true positives}_b+\textit{false positives}_b}$ for each bin *b*). Furthermore, we assemble the reads before and after binning using LRBinner. Metagenomics assemblies were performed using wtdbg2 [28] and metaFlye [29]. We compare genome fractions, CPU-time and memory usage in assembly evaluation. We used MetaQUAST [30] to obtain the average genome fraction (average over all the reference genomes) for the qualitative evaluation of assembled contigs.

6 $$\begin{aligned} Precision= & {} \frac{\sum _{k}max_s \{a_{ks}\}}{\sum _{k}\sum _{s}a_{ks}} \end{aligned}$$

7 $$\begin{aligned} Recall= & {} \frac{\sum _{s}max_k \{a_{ks}\}}{\sum _{k}\sum _{s}a_{ks}} \end{aligned}$$

8 $$\begin{aligned} F1= & {} 2\times \frac{Precision\times Recall}{Precision+Recall} \end{aligned}$$

## Results and discussion

We first compare precision, recall, F1 score and the estimated number of bins for binning performance. We further present the completeness and contamination results of bins produced by different binners. We finally evaluate assembly results using genome fraction and recorded the resource utilisation for the chosen assembly tools.

### Binning results

We benchmarked the binning performance MetaBCC-LR and LRBinner using typical metrics for evluating binning performance [15]. Table 1 demonstrates the binning results in terms of precision, recall, F1-score and the number of inferred bins. While MetaBCC-LR and LRBinner perform in a comparable fashion on simulated datasets, LRBinner achieves the best estimation on the number of bins with respect to the ground truth for most of the datasets. Note that LRBinner improves binning results for all the real datasets as indicated by the higher F1 scores.

**Fig. 3:**
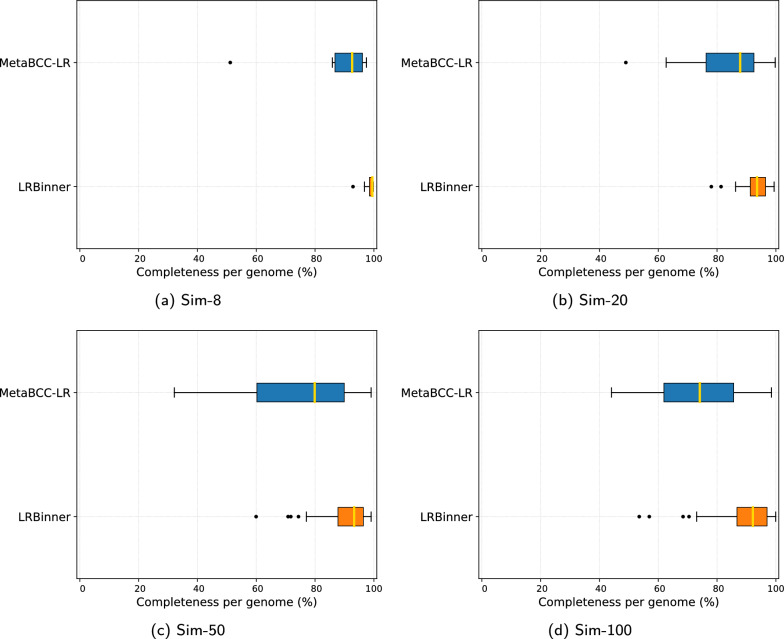
Comparison of bin completeness between MetaBCC-LR and LRBinner for the simulated datasets

**Fig. 4:**
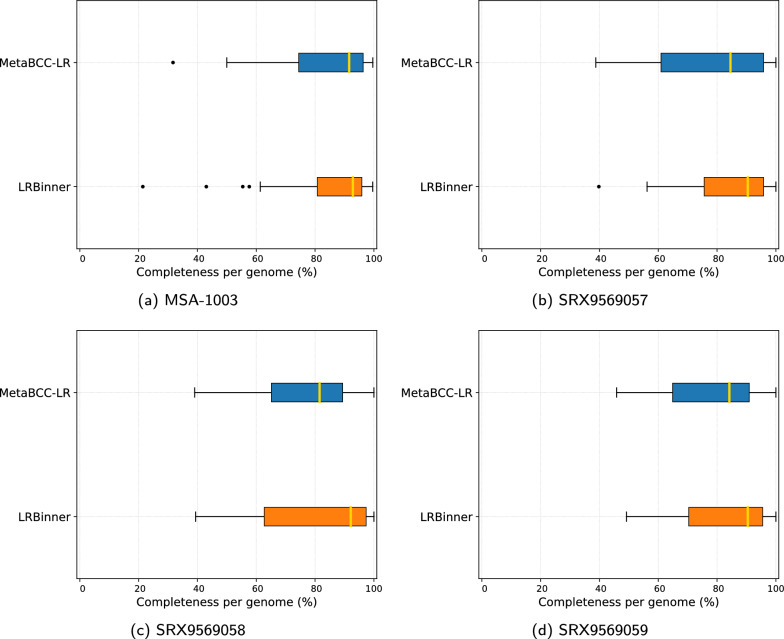
Comparison of bin completeness between MetaBCC-LR and LRBinner for the real datasets

**Fig. 5:**
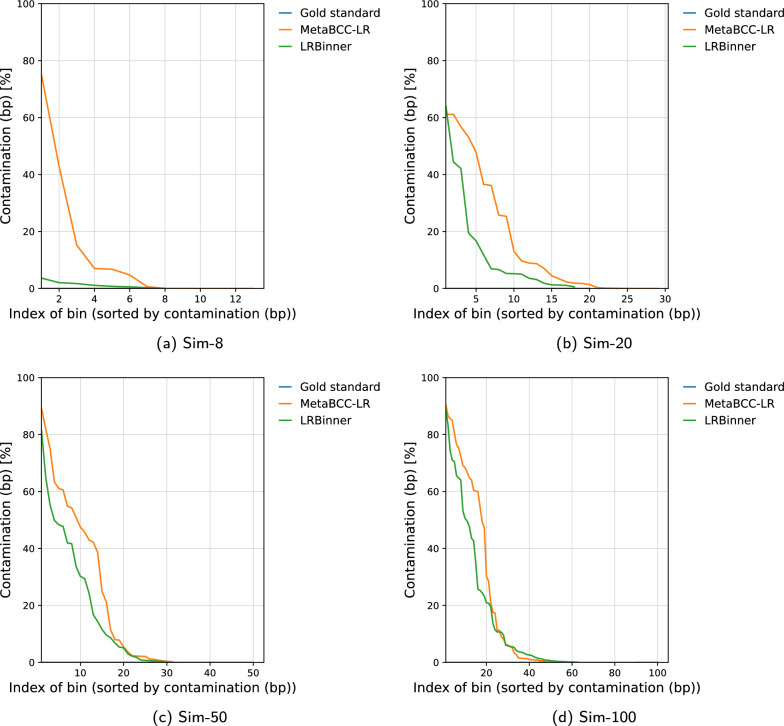
Comparison of bin contamination between MetaBCC-LR and LRBinner for the simulated datasets

**Fig. 6:**
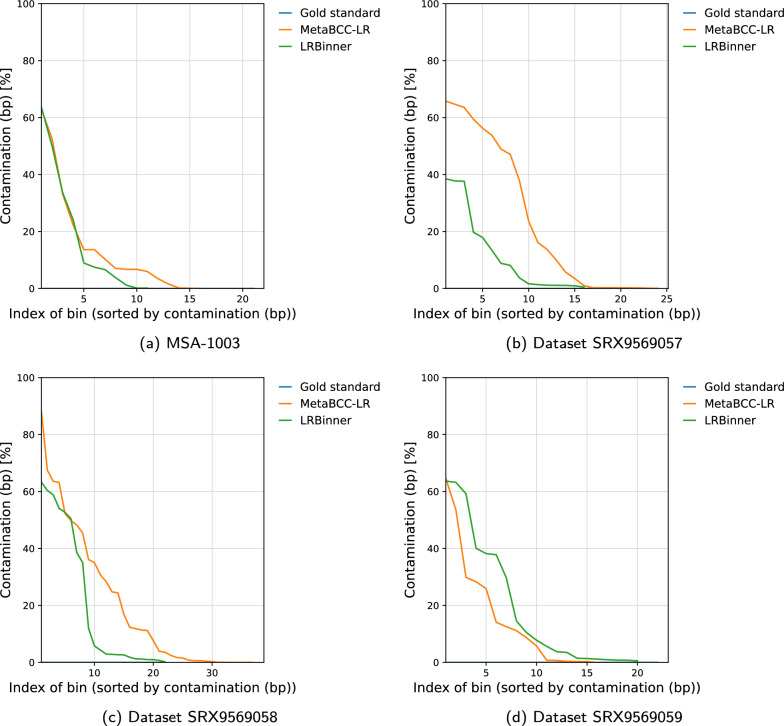
Comparison of bin contamination between MetaBCC-LR and LRBinner for the real datasets

**Fig. 7:**
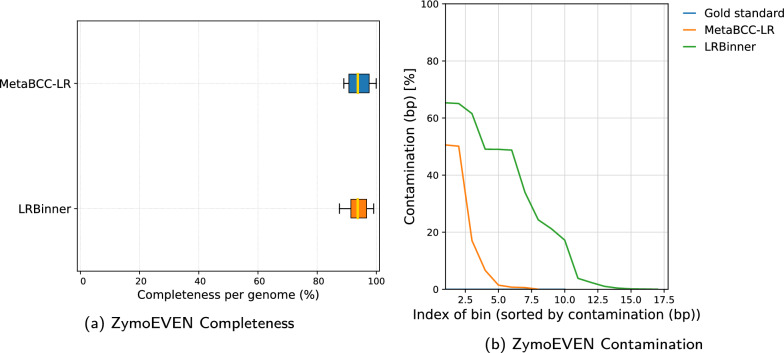
Comparison of bin completeness and contamination between MetaBCC-LR and LRBinner for the real nanopore dataset

Figures 3 and  4 illustrates the completeness of bins produced by MetaBCC-LR and LRBinner, for simulated and real datasets respectively. LRBinner has been able to produce bins with better average completeness over MetaBCC-LR. Figures 5 and 6 also illustrates the contamination levels of bins produced by MetaBCC-LR and LRBinner, for simulated and real datasets respectively. From the plots it is evident that LRBinner produces bins with lower contamination in all datasets except for **SRX9569059**. Note that the dataset **SRX9569059** has been generated from a PCR amplified sample leading to a significant deviation from the original sample abundances in contrast with **SRX9569057** and **SRX9569058** datasets. For example, in **SRX9569059**, the abundance of *Faecalibacterium prausnitzii* drops from $\sim 16\%$ to $\sim 8\%$ whereas the abundance of *Fusobacterium nucleatum* surges from $\sim 4\%$ to $\sim 7\%$, which may result in contamination of long reads in binning results. Figure 7 illustrates completeness and contamination for binning result of **ZymoEVEN** dataset. Both tools have comparable completeness over bins. LRBinner produce 8 bins with less than 20% contamination where as MetaBCC-LR has only 5 such bins. This observation is likely due to the higher error-rate observed among nanopore reads which deviate both coverage and composition vectors from the origin species. Since coverage and composition are not combined in MetaBCC-LR, it can still perform comparable to LRBinner.

### Assembly results

We assembled the reads binned by LRBinner to evaluate the potential assembly quality changes. For the assembly, we chose the two state-of-the-art long-read assemblers wtdbg2 [28] and metaFlye [29]. Table 2, demonstrates that binning long reads prior to assembly by LRBinner improves the genome fraction for all wtdbg2 assemblies (up to 40%) and maintains comparable genome fractions for metaFlye assemblies. This is not surprising as metaFlye is a metagenomics specialised assembler in contrast with wtdbg2. For example, in the datasets SRX9569057, SRX9569058 and SRX9569059, binning via LRBinner enabled wtdbg2 to recover low-abundance species which were ignored in the assembly of the entire raw dataset, *e.g., Methanobrevibacter smithii* (from 0 to 96%), *Saccharomyces cerevisiae* (from 0 to 75%) and *Candida albican* (from 0 to 70%). This is because LRBinner allows wtdbg2 to estimate more appropriate parameters in each bin rather than applying the same parameters across the entire dataset.

Another advantage of binning prior to assembly is the reduction of the computing resources for assembly. As demonstrated in Table 2, the peak-memory usage has been drastically reduced in both wtdbg2 (upto $10\times$) and metaFlye (upto $4\times$) assemblies. Note that the CPU time is comparable as binning long reads may not lead to significant reduction of *k*-mer indexing cost and the construction and simplification of assembly graphs.

## Implementation

In order to restrict the iterative search for clusters, we use early termination parameters in our algorithm. We stop drawing seed points when the remaining number of reads reaches below $min\_cluster\_size$ (=5000 by default) or the number of iterations has passed $max\_iterations$ (=1000 by default). In order to evaluate the performance of with varying size of the composition vectors (*k*-mer size) we executed LRBinner with *k*=3, *k*=4 and *k*=5. The resource utilization and performance are shown in 1: Tables S3 and S4. The GPU utilization was below 4GB during all the experiments due to fixed batch size of 1024 reads. Coverage vectors were fixed at $bins$ $=$ $32$ and $bin\_width$ $=$ $10$. Furthermore, the parameters for training auto-encoder, *i.e.*, loss function weights, were empirically determined based on the intuition of giving more prominence towards clustering. Hence, we have weighted composition more than coverage as composition (computed from smaller *k*-mer sizes) is usually more accurate compared to coverage (estimated from larger *k*-mer sizes). Furthermore, we have weighed the KL-divergence the least, to facilitate learning of disentangled representations enabling clustering.

LRBinner was implemented using C++ and Python version 3.7. The deep learning component is implemented using PyTorch [31] and Numpy [32]. We conducted our assemblies on NCI Australia with 2 x 28-core Intel Xeon Platinum 8274 (Cascade Lake) 3.2 GHz CPUs 192GB RAM and binning on Ubuntu 20.04.3 LTS system running on AMD Ryzen 9 5950X with 16-core Processor with 32 threads and 128GB of RAM with NVIDIA RTX 3090 GPU with 24GB VRAM. We used 56 threads for assembly and 32 threads for binning with GPU acceleration.

## Conclusion

In this paper, we presented LRBinner, a long read binner capable of binning error-prone long reads using both coverage and composition information. Our work extends the use of variational auto-encoders to combine raw features and learn a better latent representation for long-read binning. Furthermore, we presented a novel clustering strategy that can perform clustering on large datasets with varying cluster sizes. Performance of LRBinner was evaluated against existing long-read binners using simulated and real datasets. Our experimental results show that LRBinner outperforms state-of-the-art long-read binning tools and also improves resource usage of downstream assembly.

However, LRBinner still suffers from the following limitations. Firstly, it is challenging for LRBinner to distinguish long reads from similar regions shared between different species. Reads from such regions are likely to be assigned to any one of the species because LRBinner currently does not support overlapped binning. Similar to GraphBin2 [33], LRBinner can be extended to detect such long reads and improve on the functionality of overlapped binning among distinct species. Secondly, LRBinner uses the valley-to-peak ratio to find candidate clusters which depends on the seed points. We intend to extend LRBinner to incorporate cluster scoring techniques such as Silhouette score to automatically find best candidate clusters. Thirdly, while the use of LRBinner prior to assembly increases the genome fraction, it may also result in a more fragmented assemblies (refer to 1: Table S5 in the Additional material). Hence, we are keen to explore the possibility of combining binning and assembly of long reads simultaneously in the future.

## Supplementary Information

**Additional file 1.** Information about datasets. LR_Binner_BMC___Supp_ESM.pdf. Evaluation of LRBinner for varying composition k-mer sizes. Extended assembly quality evaluation.
