# Supplementary material for: Binning long reads in metagenomics datasets using composition and coverage information
Source: Algorithms Mol Biol. 2022 Jul 11;17:14. doi: 10.1186/s13015-022-00221-z (PMC9277797; doi:10.1186/s13015-022-00221-z)
Supplement: Supplementary file 1 — Additional file 1. Information about datasets. LR_Binner_BMC___Supp_ESM.pdf. Evaluation of LRBinner for varying composition k-mer sizes. Extended assembly quality evaluation. [file 13015_2022_221_MOESM1_ESM.pdf]

Additional file 1 for "Binning Long Reads in Metagenomics Datasets Using Composition and Coverage Information"

Anuradha Wickramarachchi and Yu Lin

1 Information About Datasets

Tables 1 and 2 demonstrate the simulated and real dataset information respectively. Note that the Table 1 tabulates the coverages used for simulation using SimLoRD [1]. Table 2 tabulates the abundances from the original data sources.

Dataset	Number of Reads	Total Size	Species	Coverage
Sim-8	432,333	3.5GB	Acetobacter pasteurianus	25
			Bacillus cereus	50
			Chlamydophila psittaci	80
			Escherichia coli	125
			Haemophilus parainfluenzae	350
			Lactobacillus casei	200
			Thermococcus sibiricus	150
			Streptomyces scabiei	100
Sim-20	666,735	5.3Gb	Amycolatopsis mediterranei	25
			Arthrobacter arilaitensis	65
			Brachyspira intermedia	20
			Corynebacterium ulcerans	40
			Erysipelothrix rhusiopathiae	55
			Enterococcus faecium	50
			Mycobacterium bovis	80
			Photobacterium profundum	85
			Streptococcus pyogenes	100
			Xanthobacter autotrophicus	150
			Rhizobium leguminosarum	100
			Francisella novicida	150
			Candidatus Pelagibacter ubique	67
			Halobacterium sp	65
			Lactobacillus delbrueckii	60
			Paenibacillus mucilaginosus	90
			Rickettsia prowazekii	100
			Thermoanaerobacter brockii	110
			Yersinia pestis	105
			Nitrosococcus watsonii	95

Dataset	Number of Reads	Total Size	Species	Coverage
Sim-50	1,119,439	9.5GB	Azorhizobium caulinodans	25
			Bacillus cereus	35
			Bdellovibrio bacteriovorus	21
			Bifidobacterium adolescentis	44
			Bifidobacterium animalis	31
			Campylobacter jejuni	11
			Clostridium tetani	36
			Clostridium thermocellum	31
			Corynebacterium diphtheriae	42
			Corynebacterium ulcerans	33
			Ehrlichia ruminantium	26
			Enterococcus faecium	24
			Erysipelothrix rhusiopathiae	44
			Escherichia coli	20
			Fervidicoccus fontis	49
			Francisella novicida	42
			Francisella tularensis	49
			Fusobacterium nucleatum	39
			Haemophilus influenzae	12
			Haemophilus parainfluenzae	11
			Haemophilus somnus	44
			Helicobacter pylori	47
			Hyphomicrobium sp	44
			Lawsonia intracellularis	46
			Metallosphaera cuprina	33
			Methanosarcina barkeri	44
			Micrococcus luteus	46
			Mycobacterium bovis	42
			Mycoplasma gallisepticum	29
			Neisseria meningitidis	38
			Nitrosococcus watsonii	42
			Paenibacillus mucilaginosus	14
			Paenibacillus sp	31
			Photobacterium profundum	45
			Pseudogulbenkiania sp	25
			Pseudomonas putida	10
			Rhizobium leguminosarum	20
			Rickettsia prowazekii	38
			Rickettsia rickettsii	100
			Ruegeria sp	200
			Shewanella sp	90
			Sodalis glossinidius	120
			Staphylococcus aureus	220
			Streptococcus pyogenes	110
			Streptococcus suis	100
			Streptomyces scabiei	110
			Symbiobacterium thermophilum	250
			Thermoanaerobacter sp	220
			Thermococcus sibiricus	210
			Variovorax paradoxus	100

Dataset	Number of Reads	Total Size	Species	Coverage
Sim-100	2,991,815	24.6GB	Acetobacter pasteurianus	20
			Aeromonas veronii	5
			Amycolatopsis mediterranei	5
			Arthrobacter arilaitensis	5
			Azorhizobium caulinodans	25
			Bacillus cereus	35
			Bacillus thuringiensis	5
			Bdellovibrio bacteriovorus	21
			Bifidobacterium adolescentis	44
			Bifidobacterium animalis	31
			Brachyspira intermedia	5
			Campylobacter jejuni	11
			Candidatus Pelagibacter ubique	5
			Candidatus Phytoplasma mali	10
			Candidatus Sulcia muelleri	23
			Chlamydia trachomatis	23
			Chlamydophila psittaci	23
			Clostridium acetobutylicum	16
			Clostridium botulinum	5
			Clostridium tetani	36
			Clostridium thermocellum	31
			Corynebacterium diphtheriae	42
			Corynebacterium pseudotuberculosis	5
			Corynebacterium ulcerans	33
			Cyanobacterium UCYN	5
			Cyanothece sp	5
			Desulfovibrio vulgaris	17
			Ehrlichia ruminantium	26
			Enterococcus faecium	24
			Erysipelothrix rhusiopathiae	44
			Escherichia coli	20
			Fervidicoccus fontis	49
			Fibrobacter succinogenes	14
			Flavobacterium branchiophilum	9
			Francisella novicida	42
			Francisella tularensis	49
			Fusobacterium nucleatum	39
			Gardnerella vaginalis	7
			Granulicella tundricola	8
			Haemophilus influenzae	12
			Haemophilus parainfluenzae	11
			Haemophilus somnus	44
			Halobacterium sp	15
			Halothiobacillus neapolitanus	5
			Helicobacter pylori	47
			Hyphomicrobium sp	44
			Ignavibacterium album	17
			Klebsiella oxytoca	14
			Krokinobacter sp	5
			Lactobacillus brevis	9

Dataset	Number of Reads	Total Size	Species	Coverage
			Lactobacillus casei	5
			Lactobacillus delbrueckii	19
			Lawsonia intracellularis	46
			Legionella pneumophila	5
			Metallosphaera cuprina	33
			Methanocorpusculum labreanum	45
			Methanosarcina acetivorans	32
			Methanosarcina barkeri	44
			Micrococcus luteus	46
			Mycobacterium bovis	42
			Mycobacterium sp	30
			Mycoplasma gallisepticum	29
			Mycoplasma hyorhinis	34
			Neisseria meningitidis	38
			Nitrosococcus watsonii	42
			Nitrosomonas sp	5
			Nocardia farcinica	5
			Odoribacter splanchnicus	11
			Paenibacillus mucilaginosus	14
			Paenibacillus sp	31
			Photobacterium profundum	45
			Prochlorococcus marinus	5
			Pseudogulbenkiania sp	25
			Pseudomonas putida	10
			Rhizobium leguminosarum	20
			Rhodococcus jostii	5
			Rickettsia prowazekii	38
			Rickettsia rickettsii	45
			Rickettsia slovaca	5
			Ruegeria sp	30
			Salmonella enterica	5
			Sebaldella termitidis	8
			Shewanella sp	30
			Shigella flexneri	5
			Sodalis glossinidius	27
			Staphylococcus aureus	40
			Streptococcus pneumoniae	7
			Streptococcus pyogenes	10
			Streptococcus suis	48
			Streptococcus thermophilus	5
			Streptomyces scabiei	20
			Symbiobacterium thermophilum	37
			Thermoanaerobacter brockii	5
			Thermoanaerobacter sp	23
			Thermococcus sibiricus	38
			Variovorax paradoxus	34
			Weeksella virosa	5
			Wolbachia sp	43
			Xanthobacter autotrophicus	44
			Yersinia pestis	9

Table 1: Information of simulated datasets.

Dataset	Number of Reads	Total Size	Species	Abundance
ZymoEVEN	1,688,672	8.2Gb	Pseudomonas aeruginosa	9.7%
			Escherichia coli	9.9%
			Salmonella enterica	10.0%
			Lactobacillus fermentum	9.3%
			Enterococcus faecalis	12.2%
			Staphylococcus aureus	11.2%
			Listeria monocytogenes	14.5%
			Bacillus subtilis	19.3%
			Saccharomyces cerevisiae	2.1%
			Cryptococcus neoformans	1.8%
MSA-1003	2,358,257	19GB	Acinetobacter baumannii	0.18%
			Bacillus pacificus	1.80%
			Bacteroides vulgatus	0.02%
			Bifidobacterium adolescentis	0.02%
			Clostridium beijerinckii	1.80%
			Cutibacterium acnes	0.18%
			Deinococcus radiodurans	0.02%
			Enterococcus faecalis	0.02%
			Escherichia coli	18.0%
			Helicobacter pylori	0.18%
			Lactobacillus gasseri	0.18%
			Neisseria meningitidis	0.18%
			Porphyromonas gingivalis	18.0%
			Pseudomonas aeruginosa	1.80%
			Rhodobacter sphaeroides	18.0%
			Schaalia odontolytica	0.02%
			Staphylococcus aureus	1.80%
			Staphylococcus epidermidis	18.0%
			Streptococcus agalactiae	1.80%
			Streptococcus mutans	18.0%
SRX9569057	1,978,852	17GB	Faecalibacterium prausnitzii	14.82%
SRX9569058	2,770,833	25GB	Veillonella rogosae	20.01%
SRX9569059	2,480,208	20GB	Roseburia hominis	12.47%
			Bacteroides fragilis	8.36%
			Prevotella corporis	6.28%
			Bifidobacterium adolescentis	8.86%
			Fusobacterium nucleatum	7.56%
			Lactobacillus fermentum	9.71%
			Clostridioides difficile	1.10%
			Akkermansia muciniphila	1.62%
			Methanobrevibacter smithii	0.17%
			Salmonella enterica	0.0065%
			Enterococcus faecalis	0.0011%
			Clostridium perfringens	0.00009%
			Escherichia coli (JM109)	1.83%
			Escherichia coli (B-3008)	1.82%
			Escherichia coli (B-2207)	1.65%
			Escherichia coli (B-766)	1.66%
			Escherichia coli (B-1109)	1.77%
			Candida albicans	0.16%
			Saccharomyces cerevisiae	0.16%

Table 2: Information of real datasets.

2 Evaluation of LRBinner for varying composition k-mer sizes

Table 3 and 4 demonstrate the resource consumption and binning performance for varying k-mer sizes.

Dataset	k-size	CPU Hours	Peak Memory (GB)
Sim-8	3	**0.72**	**4.52**
	4	0.79	5.51
	5	0.79	9.29
Sim-20	3	**1.06**	**4.83**
	4	1.11	6.34
	5	1.14	12.16
Sim-50	3	**1.70**	**5.46**
	4	1.77	7.96
	5	1.82	19.06
Sim-100	3	**4.12**	**7.95**
	4	4.39	14.59
	5	4.37	42.27
ZymoEVEN	3	**1.87**	**6.23**
	4	2.09	10.00
	5	2.03	24.85
MSA-1003	3	**3.31**	**7.09**
	4	3.38	12.35
	5	3.49	33.70
SRX9569057	3	**2.98**	**6.59**
	4	3.03	11.00
	5	3.14	28.56
SRX9569058	3	**1.65**	**7.66**
	4	2.34	13.81
	5	4.41	39.28
SRX9569059	3	**1.62**	**7.25**
	4	2.46	12.76
	5	3.90	35.32

Table 3: Run time and memory consumption of LRBinner with varying k-sizes for composition vectors.

Dataset	k-size	No. of Bins	Precision	Recall	F1 score
Sim-8	3	**8**	**99.14%**	**99.14%**	**99.14%**
	4	10	97.22%	96.25%	96.73%
	5	10	97.18%	98.47%	97.82%
Sim-20	3	18	90.53%	88.23%	89.36%
	4	**20**	91.58%	91.58%	91.58%
	5	21	**93.11%**	**95.96%**	**94.51%**
Sim-50	3	31	82.60%	**92.78%**	87.39%
	4	42	92.78%	87.00%	89.79%
	5	**45**	**93.72%**	88.63%	**91.11%**
Sim-100	3	**63**	82.60%	**92.78%**	**87.39%**
	4	57	92.87%	82.13%	87.17%
	5	55	**93.22%**	81.05%	86.71%
ZymoEVEN	3	17	**91.26%**	75.36%	82.55%
	4	6	90.09%	46.99%	61.76%
	5	**13**	89.35%	**85.74%**	**87.51%**
MSA-1003	3	**10**	**95.30%**	95.99%	95.64%
	4	23	91.71%	96.66%	94.12%
	5	33	94.84%	**97.72%**	**96.26%**
SRX9569057	3	**16**	80.47%	90.68%	85.27%
	4	30	**82.07%**	90.92%	86.27%
	5	38	80.18%	**95.07%**	**86.99%**
SRX9569058	3	22	73.72%	91.03%	81.46%
	4	30	**81.88%**	90.80%	86.11%
	5	42	80.44%	**95.80%**	**87.45%**
SRX9569059	3	**20**	79.70%	91.25%	85.08%
	4	29	**83.18%**	89.42%	**86.19%**
	5	56	77.94%	**96.02%**	86.04%

Table 4: Performance of LRBinner with varying k-sizes for composition vectors.

3 Extended assembly quality evaluation

Table 3 demonstrates in-depth assembly quality evaluation for **Sim-8** dataset.

Metric	metaFlye Without Binning	metaFlye With Binning	wtdbg2 Without Binning	wtdbg2 With Binning
Genome Fraction	99.90%	99.85%	98.80%	98.90%
Largest contig	6462189	6458057	10112088	2332397
Total length	31422943	32346521	31202217	32497405
NA50	2761858	1845135	2740614	750161
N50	2763817	1845136	2952899	750180

Table 5: Extended assembly quality evaluation for **Sim-8** dataset.

References

[1] Bianca K. Stöcker, Johannes Köster, and Sven Rahmann. SimLoRD: Simulation of Long Read Data. *Bioinformatics*, 32(17):2704–2706, 05 2016.
